# Dementia in Parkinson's Disease Correlates with ***α***-Synuclein Pathology but Not with Cortical Astrogliosis

**DOI:** 10.1155/2012/420957

**Published:** 2012-04-22

**Authors:** Simone A. van den Berge, Josta T. Kevenaar, Jacqueline A. Sluijs, Elly M. Hol

**Affiliations:** ^1^Department of Astrocyte Biology & Neurodegeneration, Netherlands Institute for Neuroscience (NIN), Royal Netherlands Academy of Arts and Sciences, 1105 BA Amsterdam, The Netherlands; ^2^Center for Neuroscience Swammerdam Institute for Life Sciences, University of Amsterdam, 1098 XH Amsterdam, The Netherlands

## Abstract

Dementia is a common feature in Parkinson's disease (PD) and is considered to be the result of limbic and cortical Lewy bodies and/or Alzheimer changes. Astrogliosis may also affect the development of dementia, since it correlates well with declining cognition in Alzheimer patients. Thus, we determined whether cortical astrogliosis occurs in PD, whether it is related to dementia, and whether this is reflected by the presence of glial fibrillary acidic protein (GFAP) and vimentin in cerebrospinal fluid (CSF). We have examined these proteins by immunohistochemistry in the frontal cortex and by Western blot in CSF of cases with PD, PD with dementia (PDD), dementia with Lewy bodies (DLB) and nondemented controls. We were neither able to detect an increase in cortical astrogliosis in PD, PDD, or DLB nor could we observe a correlation between the extent of astrogliosis and the degree of dementia. The levels of GFAP and vimentin in CSF did not correlate to the extent of astrogliosis or dementia. We did confirm the previously identified positive correlation between the presence of cortical Lewy bodies and dementia in PD. In conclusion, we have shown that cortical astrogliosis is not associated with the cognitive decline in Lewy body-related dementia.

## 1. Introduction

Parkinson's disease (PD) is a progressive neurological disorder characterized by motor symptoms such as tremor, bradykinesia, rigidity, and postural instability. It is associated with an almost complete degenerative loss of dopamine neurons in the substantia nigra (SN) pars compacta and the presence of Lewy bodies (LBs) and Lewy neurites (LNs). The latter pathological hallmarks initially occur, according to Braak and colleagues, in the glossopharyngeal and vagal nerves and the anterior olfactory nucleus, and thereafter spread to other brain nuclei and cortical areas [1]. This topological progression of the disease, however, is currently being critically evaluated in the field [2–4]. In any case, a major component of these LBs and LNs is an aggregated form of the presynaptic protein *α*-synuclein [1].

More recently, it has become apparent that cognitive dysfunction is also an important clinical component of PD. About two-third of the patients develop cognitive deficits within 3.5 years from the disease onset [5], and up to 40% of idiopathic PD patients will develop dementia (PD with dementia, PDD) based on population-based studies (reviewed in [6]). The cognitive deficits and dementia are considered to be the result of the limbic and cortical LBs [5], although the presence of Alzheimer changes, such as neurofibrillary tangles and amyloid depositions may also play a role in some elderly PD patients. LBs are not specific for PD and are also found in dementia with Lewy bodies (DLBs). DLB is currently considered to be part of a spectrum of dementias related to cortical LB disease, instead of a specific disease entity, as neither the profile of cognitive deficits nor the amount of LB pathology allow differentiation between DLB and PDD (reviewed in [7]). The dementia seen in PDD and DLB is typically a deficit in executive functions, resembling the symptoms seen in frontal lobe patients (reviewed in [6]). A number of neuropathological and neurochemical changes have been linked to LB-related dementias, amongst which cholinergic deficiency, Alzheimer's disease (AD) pathology, and cortical LBs. However, there is no association between the number of cortical LBs and the timing of dementia, which suggests that there may be other factors involved [7]. A factor that has not been investigated with regard to the development of cognitive changes is astrogliosis, which is an interesting candidate as it has already been shown to be correlated to the cognitive status in AD patients [8].

Astrocytes have many functions in the CNS (recently reviewed by [9–11]), such as regulation of local vasodilation and blood flow, provision of energy metabolites to neurons, participation in synaptic function and plasticity, modulation of neuronal communication, and maintenance of the extracellular balance of ions, fluids, and transmitters. In response to CNS injury or during the course of a neurodegenerative disease, astrocytes become reactive, which is characterized by hypertrophy of cellular processes and upregulation of the intermediate filament proteins vimentin, GFAP, nestin, and synemin [12]. Depending on the context of activation, reactive astrocytes are involved in neuronal survival and regeneration in either a protective or impedimental way (reviewed in [13]). In AD, the extent of astrogliosis, as shown by GFAP levels in both cortex and cerebrospinal fluid (CSF) is correlated to the degree of dementia [14, 15]. This suggests that the dementia might be partly due to a pathological modification in the function of astrocytes instigated by reactive gliosis, leading to a change in their synapse modulating activities.

Scientific literature on astrogliosis in PD is limited, and contradicting data have been reported. In rodent models, induced by toxins, reactive astrocytes are observed in the first days after model induction [17, 18]. In the 1-methyl-4-phenyl-1,2,3,6-tetrahydropyridine (MPTP) model, the astrocytic reaction in the SN and striatum follows microglia activation and parallels the time course of dopaminergic cell death in the SN. In these animals, the GFAP expression remains upregulated, even after most dopaminergic neurons have died due to MPTP intoxication [18]. In the 6-hydroxy dopamine (6-OHDA) model, GFAP upregulation is transient and returns to control levels after 28 days [17]. Furthermore, 6-OHDA injection in the SN or medial forebrain bundle can induce GFAP changes over long distances in the striatum and even in the cortex [17, 19]. In the human brain, inconsistent results have been found; in two studies no evidence for GFAP upregulation in the SN and putamen of PD patients was found [20, 21], other studies, however, did show the presence of reactive astrocytes, and thus an increase in GFAP, in the SN [22–25]. In addition, astrocytes in the parkinsonian brain upregulate DnaJB6, a protein that is also found in LBs [26]. These contradicting studies and the lack of information on astrogliosis in PD-related dementias led us to question whether the frontal-type dementia in PD is related to astrogliosis in the frontal lobe of PD brains. We chose to study the frontal cortex both because the dementia in PD presents with frontal deficits [6], and because LB load in the frontal cortex is an indicator of cognitive impairment in PD patients [27]. We hypothesized that reactive astrocytes in frontal cortical areas of PD brains are associated with the occurrence of dementia, and that this is reflected in GFAP and vimentin levels in the CSF. If this is true, the astrocytic CSF markers can be potentially used as a diagnostic indicator for astrocyte dysfunction and dementia. In addition, we investigated *α*-synuclein pathology in the frontal cortex to determine its correlation to dementia in the PDD and DLB cases studied.

## 2. Materials and Methods

### 2.1. Brain Tissue and Donor Selection

Human postmortem tissue was obtained from the Netherlands Brain Bank (NBB; Amsterdam, The Netherlands). The NBB performs rapid brain autopsies, and the brain donors have given written informed consent for using the tissue and for accessing the extensive neuropathological and clinical information for scientific research, in compliance with ethical and legal guidelines [31]. All autopsies are performed at the designated premises of the VU University medical center in Amsterdam. Diagnostic examination and dissection of the central nervous system organs are performed by pathologists registered in accordance with Individual Health Care Professions Act. The independent Review Board of the VU University medical center has reviewed and agreed with the procedures of the Netherlands Brain Bank concerning “donation of brain material for scientific research”. Two brain donors in our cohort requested euthanasia as they suffered from metastasized carcinoma (see Table 2). According to Dutch law, euthanasia is legal. The drugs to induce euthanasia in donor 2001-046 were a cocktail of fentanyl (an opiate agonist), midazolam (a benzodiazepine), thiopental (rapid-onset short-acting barbiturate general anaesthetic), and pancuronium (a muscle relaxant). Donor 2004-045 passed away after receiving thiopental and pancuronium. Extensive neuropathological examinations show that the brains of these cases were not metastasized by the tumour. One patient had a small subependymoma, but this was clinically irrelevant according to the neuropathologist. Furthermore, GFAP and vimentin expression of these donors was not markedly increased or decreased as compared to the other donors. Therefore, it is unlikely that the drugs by which euthanasia was induced affected our data.

For this study, we selected 40 cases, divided into four diagnostic groups (see details in Table 1) based on the clinical and pathological data from the NBB. The first group consisted of 10 neurological control cases, for which the clinical files did not report a history of neurological diseases, cognitive problems or memory deficits. In addition, these cases did not show Lewy body pathology. The second group included 9 cases diagnosed with PD without reported memory problems or dementia. The third group included 11 cases diagnosed with PDD, and the last group consisted of 10 cases diagnosed with DLB. The diagnosis PDD is given to cases that present first with PD and develop dementia after at least a year, while DLB patients show cognitive problems first [7]. Groups were matched for sex, age (*P* = 0.301), brain weight (*P* = 0.284), and postmortem delay (PMD; *P* = 0.229) (see Table 1). Only cases with an Alzheimer score [28] of Braak 0 or 1 were included, assuring the absence of tangles in the frontal cortex and, therefore, excluding these Alzheimer changes as the primary cause of the dementia. The amyloid deposition was quite variable, and scores between O (no amyloid deposition) and C (extensive amyloid deposition) were present in each group. A score for dementia (Reisberg scale/global deterioration scale; GDS [16]) was available for 8 out of 11 PDD cases and 9 out of 10 DLB cases. A GDS score of 1 implies no cognitive decline; a GDS score of 7 implies a very severe cognitive decline, that is, dementia. No GDS scores were available of the control and PD group, but the clinical history indicated normal cognitive functioning. These cases were, therefore, excluded from tests in which GDS scores were analyzed. Comparing the mean GDS scores between the DLB group and the PDD group showed a significant difference between the two groups (*P* = 0.004), with a higher median GDS score (median = 7.00) in cases with LBD than cases with PDD (median = 5.00). All other donor parameters, such as amyloid pathology and ApoE genotype (see also Tables 1 and 2), were not significantly different between the groups.

PD case NBB 2002-105 had unusual high scores of both GFAP levels in CSF (intensity = 119.8; median = 4.36) and the number of vimentin-positive cells in cortical grey matter (5676 cells/mm^3^; median = 864.5). This donor died after a stroke, which caused damage to the frontal lobe. Therefore, we have excluded this case from further analysis.

From almost all of the 40 cases, both frontal lobe sections and CSF were available for analysis. We obtained paraffin-embedded orbitofrontal or medial frontal lobe sections for 35 of the 40 cases (Table 2), which were used for immunohistochemical analysis. Postmortem CSF samples, obtained by the NBB at autopsy, were available for 37 of the 40 cases (Table 2) and were stored at −80°C until Western blot analysis.

### 2.2. Immunohistochemistry

8 *μ*m-thick sections of formalin-fixed, paraffin-embedded frontal cortex were stained for GFAP and vimentin as described before [32]. In brief, the sections were steamed in Tris-buffered saline (TBS: 0.025 M Tris, 0.14 M NaCl, pH 7.6, and 98°C), to provide optimal antigen retrieval. Then, they were preincubated in horse serum-containing buffer, and incubated overnight in the primary antibody solution (GFAP 1 : 1000, DAKO Z0334, DAKO A/S, Glostrup, Denmark; vimentin 1 : 5000, Chemicon AB5733, Millipore, Billerica, MA, USA) for 16–24 hours. Subsequently, sections were incubated with biotinylated secondary antibodies, avidin-biotin complex (ABC), and diaminobenzidine tetrahydrochloride (DAB) with nickel sulfate (NiSO_4_; Sigma-Aldrich, St. Louis, MO, USA).

*α*-synuclein immunostaining was performed according to the NBB standard protocol. Deparaffinized frontal cortex sections were brought to a boil in citrate buffer (9.4 mM citric acid, 40 mM sodium citrate, and pH 6.0) and then boiled for 10 minutes. After cooling, they were washed twice in water and incubated in 80% formic acid for 5 minutes. Then, they were washed twice in water and once in phosphate buffered saline (PBS; 137 mM NaCl, 2.7 mM KCl, 10 mM Na_2_HPO_4_, 2 mM KH_2_PO_4_, and pH 7.4), before incubation with anti-*α*-synuclein antibody (1 : 500; NCL-L-ASYN, Novocastra, Newcastle upon Tyne, UK) in PBS with 1% BSA. Sections were incubated with this antibody for two hours at room temperature and 16 hours at 4°C and then washed twice with PBS. Immunoreactivity was visualized using the EnVision + Dual Link System-HRP (DAKO): sections were incubated in Labeled Polymer-HRP for 1 hour, washed twice, and then incubated for 10 minutes in liquid DAB+ substrate (DAKO). Then, they were washed in water, dehydrated, and embedded.

### 2.3. Image Acquisition and Analysis

Images of single immunostainings were obtained on an AxioSkop microscope (Zeiss, Oberkochen, Germany) with Neoplanfluor objectives, using a Sony XC77 black and white camera (Sony, Japan) and ImagePro software (MediaCybernetics, Bethesda, USA).

For quantification of GFAP immunoreactivity, five images were taken, at 20x magnification, at random locations in grey and white matter separately. The pial layer was excluded from analysis. In the images, the background intensity was measured and a threshold of 1.7 times the background was set to measure the immunopositive surface area. Immunoreactive blood vessels and nonspecific staining were edited out before quantification. For each area the surface percentage occupied with GFAP-positive immunostaining was estimated by dividing the surface of the GFAP-positive area by the total area analyzed. Additionally, for each section the five areas were averaged for both white and grey matter regions.

For the vimentin analysis, no automated image analysis could be conducted, due to the high incidence of blood vessel staining. Therefore, vimentin-positive cells were manually identified in 15 randomly selected white and adjacent grey matter areas, which were photographed at 20x magnification. Again, the pial layer was excluded from analysis. Vimentin-positive astrocytes were identified based on their characteristic morphology. Average vimentin-positive cell densities per mm^3^ for both regions were calculated by dividing the cell counts by the total volume measured. For quantification of *α*-synuclein pathology, whole sections were examined, and the total number of LBs was counted. In addition, the surface area of the section was measured, which was used to correct LB numbers for section volume.

### 2.4. Immunoblotting

CSF of the previously described 37 cases was obtained from the NBB. Samples were prepared by mixing 10 *μ*L CSF with 10 *μ*L 20x SDS loading buffer, containing Bromophenol blue and 1 *μ*L of dithiothreitol (DTT; 2 M). Samples were heated at 95°C for 5 minutes and loaded onto 7.5% polyacrylamide gels for Western blotting. The proteins were transferred to a nitrocellulose membrane with the use of a semidry blotter. After incubating in Supermix (3.8 mM Tris; 15 mM NaCl; 60 mM Gelatin; 0.5% Triton X-100; pH 7.4) for 15 minutes, the nitrocellulose blot was incubated with anti-GFAP (1 : 50.000, DAKO, recognizing all isoforms of GFAP [33]) and anti-vimentin (1 : 7000, Chemicon) overnight at 4°C. After rinsing three times in TBS with 0.05% Tween 20 (TBS-T), the antibody/antigen complexes were identified by incubating the blots with anti-rabbit IRdye 800 (1 : 5000; Rockland) and anti-chicken Cy5 (1 : 2000) for 1 hour at room temperature. After washing in TBS-T for 3 times, protein bands were analyzed for intensity and corrected for background intensity with an Odyssey Infrared Imaging System (LI-COR Biosciences, Lincoln, USA).

### 2.5. Statistical Analysis

For statistical analysis, the program SPSS Inc 17.0 was used. All tests performed were nonparametric analyses, as most of our data was not normally distributed. We used the Mann-Whitney test for comparison of two groups and the Kruskall-Wallis test for multiple groups. Correlation analysis was performed using the Spearman test. Statistical significance was accepted when *P* ≤ 0.05.

## 3. Results

### 3.1. Astrogliosis Is Not Correlated with LB-Related Neuropathology or Dementia

**Figure 1:**
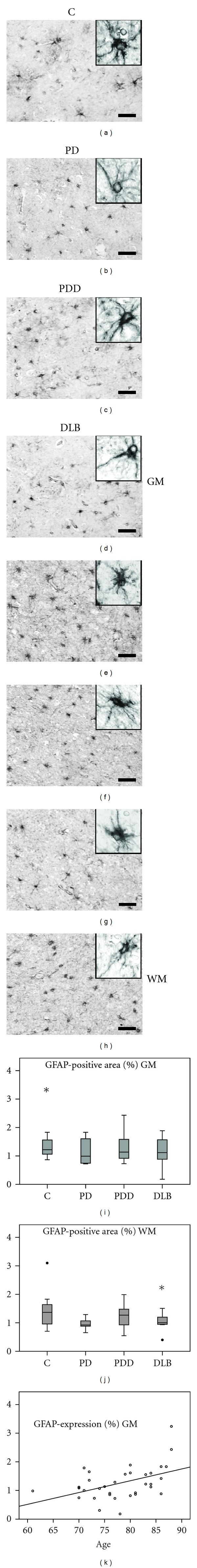
GFAP immunoreactivity in the human frontal cortex. (a–h) examples of GFAP immunoreactivity in grey (a–d) and white matter (e–h) of a 77-year-old female control (C) donor (NBB 2001-104; a, e), a 77-year-old male Parkinson's disease (PD) patient (NBB 2005-069; b, f), a 71-year-old male Parkinson's disease with dementia (PDD) patient (NBB 2004-045; c, g), and 76-year-old female dementia with Lewy bodies (DLB) patient (2001-025; d, h). (i-j) Boxplot of the quantification of GFAP staining in grey (i) and white matter (j) of 8 C, 6 PD, 11 PDD, and 9 DLB cases. (k) correlation of GFAP expression in the grey matter with age of the 34 donors. GM: grey matter; WM: white matter; inserts show higher magnification images of the same patients. Scale bars represent 50 *μ*m for the lower magnification and 20 *μ*m for the higher magnification images.

**Figure 2:**
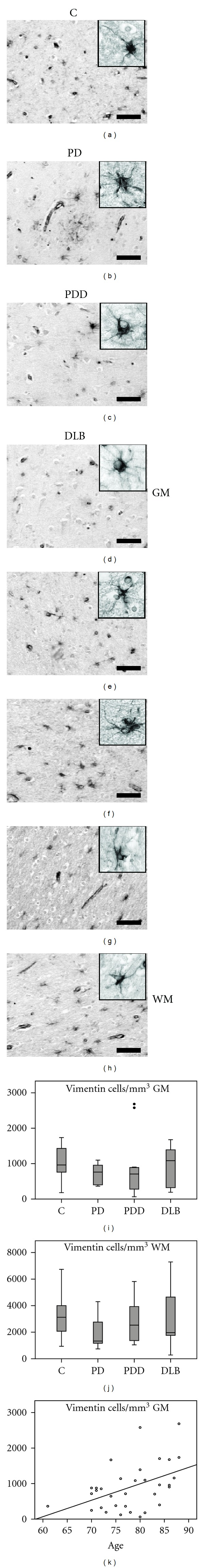
Vimentin immunoreactivity in the human frontal cortex. (a–h) examples of vimentin immunoreactivity in grey (a–d) and white matter (e–h) of a 71-year-old male control (C) donor (NBB 2002-087; a, e), a 86-year-old male Parkinson's disease (PD) patient (NBB 2001-122; b, f), a 75-year-old female Parkinson's disease with dementia (PDD) patient (NBB 2003-059; c, g), and 72-year-old male dementia with Lewy bodies (DLB) patient (2002-017; d, h). (i-j) Boxplot of the quantification of vimentin staining in grey (i) and white matter (j) of 8 C, 6 PD, 11 PDD, and 9 DLB cases. Outliers are shown as dots. (k) correlation of vimentin expression in the grey matter with age of the 34 donors. GM: grey matter; WM: white matter; inserts show higher magnification images of the same patients. Scale bars represent 50 *μ*m for the lower magnification and 20 *μ*m for the higher magnification images.

Astrocytes were abundant in both grey and white matter of the frontal cortex. Morphology of GFAP-positive astrocytes and the intensity of the staining did not differ much between grey (Figures 1(a)–1(d)) and white matter (Figures 1(e)–1(h); *P* = 0.778). The number of vimentin-positive astrocytes was higher in white matter (Figures 2(e)–2(h), median = 2533.0) than in grey matter (Figures 2(a)–2(d), median = 853.7, *P* < 0.0001). GFAP expression in frontal grey matter correlated with that in white matter (*r* = 0.441; *P* = 0.009) as well as with vimentin cell numbers in grey matter (*r* = 0.481; *P* = 0.005). Vimentin cell numbers were also correlated in grey and white matter (*r* = 0.465; *P* = 0.006). Furthermore, we observed a positive correlation between the age of the donors and GFAP (*r* = 0.421; *P* = 0.013; Figure 1(k)) and vimentin (*r* = 0.470; *P* = 0.006; Figure 2(k)) expression in grey matter.

When comparing the four diagnostic groups, GFAP expression in both grey and white matter was not significantly different in any of the groups (Figures 1(i)-1(j), Table 3, *P* = 0.755 and *P* = 0.281). The number of vimentin-positive cells was also unaltered in the PD, PDD and DLB groups compared to controls (Figures 2(i)-2(j), Table 3, *P* = 0.590 and *P* = 0.503). In addition, we found no significant correlations between GFAP and vimentin expression and the available clinical and neuropathological data, such as sex (*P* = 0.956 for GFAP in grey matter, 0.086 for GFAP in white matter, 0.802 for vimentin in grey matter, and 0.524 for vimentin in white matter), postmortem delay (PMD; *P* = 0.165, 0.513, 0.878, and 0.473), amyloid deposition (*P* = 0.111, 0.549, 0.300, and 0.389), GDS (*P* = 0.856, 0.382, 0.090, and 0.352), and ApoE genotype (*P* = 0.266, 0.942, 0.604, and 0.599).

### 3.2. GFAP and Vimentin Levels in CSF

**Figure 3:**
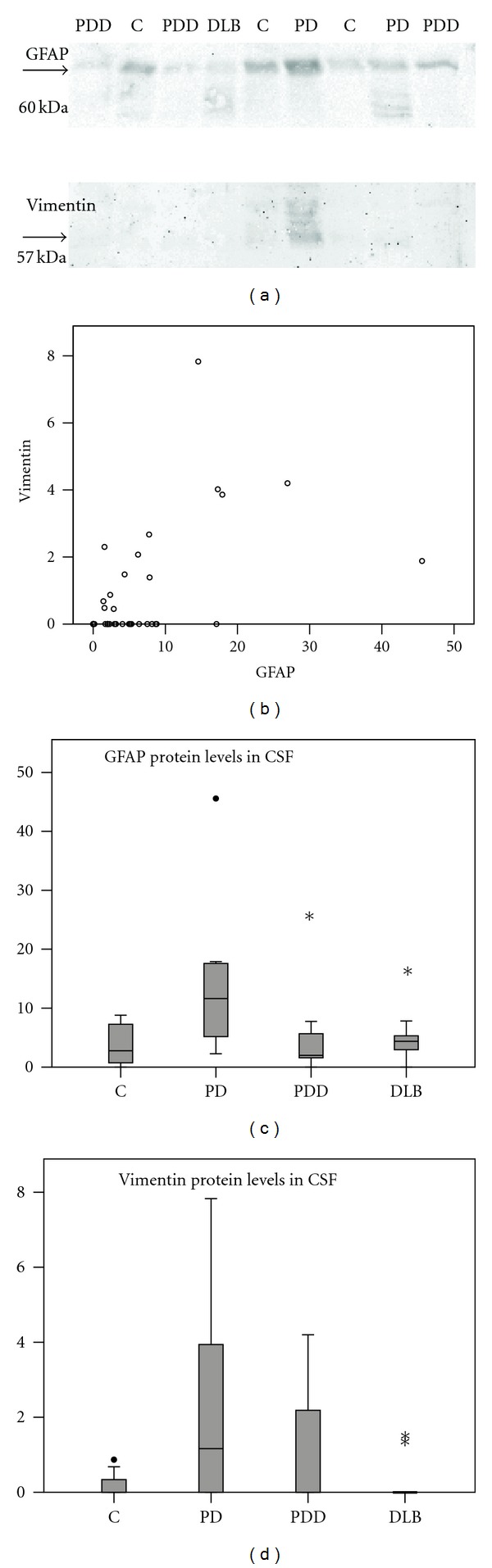
Astrocytic protein levels in ventricular cerebrospinal fluid (CSF). (a) example of a Western blot for GFAP (top row) and vimentin (bottom row) on CSF of different donors (from left to right: NBB 2006-030, 2007-007, 2007-008, 2007-019, 2005-017, 2005-050, 2005-055, 2005-069, and 2005-077). (b) correlation of the GFAP and vimentin protein levels (in arbitrary units) in CSF of 36 donors. (c) Boxplot of GFAP protein levels in CSF of 8 control (c), 8 Parkinson's disease (PD), 11 PD with dementia (PDD), and 9 dementia with Lewy bodies (DLB) cases. (d) Boxplot of vimentin protein levels in CSF of 8 C, 8 PD, 11 PDD, and 9 DLB cases.

GFAP was readily detectable in postmortem CSF of all patient groups, vimentin was found in some donors, unrelated to diagnosis (Figure 3(a), Table 3). The protein levels were not different between the four diagnostic groups (Figures 3(c)-3(d), *P* = 0.055 for GFAP, *P* = 0.156 for vimentin) and did not correlate to GDS scores (*P* = 0.513 and 0.519). There was a significant correlation between the intensity of the GFAP and the vimentin signals on the Western blot (*r* = 0.413; *P* = 0.012; Figure 3(b)). No significant correlations between Western blot values and frontal cortex levels of GFAP were found (*P* = 0.474 for grey matter and *P* = 0.542 for white matter). For vimentin, CSF protein levels negatively correlated with grey matter immunoreactivity (*P* = 0.046), but not with white matter expression (*P* = 0.445). Therefore, the levels of GFAP and vimentin do not reflect the state of astrogliosis in the frontal cortex. Also, we did not find correlations between GFAP and vimentin protein levels and the clinicopathological parameters, such as sex (*P* = 0.903 for GFAP and 0.135 for vimentin), PMD (*P* = 0.903 and 0.166), amyloid score (*P* = 0.397 and 0.399), and ApoE genotype (*P* = 0.278 and 0.255).

### 3.3. Dementia in PD Is Correlated to Lewy Body Numbers in the Frontal Cortex

**Figure 4:**
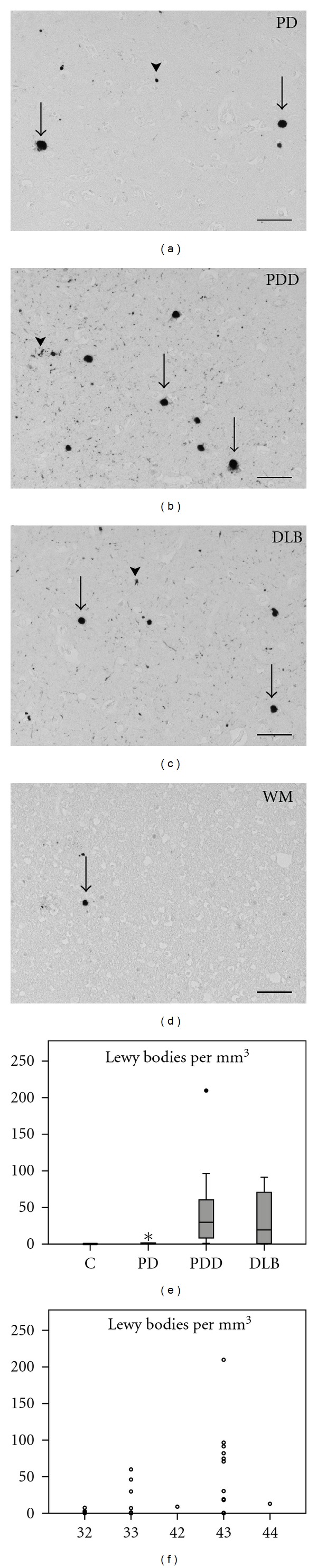
*α*-synuclein pathology in the human frontal cortex. (a–c) Lewy bodies (arrows) and Lewy neurites (arrowheads) in the grey matter of a Parkinson's disease (PD; NBB 1999-038), Parkinson's disease with dementia (PDD; NBB 2004-045), and dementia with Lewy bodies (DLB; NBB 1997-092) case. (d) a Lewy body (arrow) in the white matter (WM) of the frontal cortex of case NBB 2004-045. (e) Boxplot of the quantification of Lewy body number in the frontal cortex of 8 control, 6 PD, 11 PDD, and 9 DLB cases. (f) Lewy body numbers per ApoE genotype. Scale bars represent 100 *μ*m.

Previous studies have suggested a role of cortical LB pathology in PDD and DLB. Therefore, we quantified the number of LBs (Figures 4(a)–4(d), arrows) in the frontal cortex. LBs were observed mostly in grey matter in some of the PD cases, and in most of the PDD and DLB cases (Figures 4(a)–4(c)). We also saw LNs (Figures 4(a)–4(d), arrowheads), the presence of which correlated with the number of LBs. Occasionally, we observed some LBs in the white matter (Figure 4(d)), in PD, PDD, and DLB cases. It has to be established whether these white matter LBs are present in axons or glia cells. Astrocytes have been described to contain *α*-synuclein [21] and LBs in PD [34]. When quantifying the LB numbers (Figure 4(e), Table 3), we observed a significant difference between all groups (*P* < 0.001). When we compared the groups one by one, we found a significant difference between the control and PD groups (*P* = 0.043) and between the PD and PDD groups (*P* = 0.002). PD Braak stages were known for half of the cases in our group, and LB pathology correlated highly with these (*r* = 0.727, *P* < 0.0001), as expected. In addition, LB pathology was significantly different depending on the ApoE genotype (Figure 4(f), *P* = 0.041). When we analyzed the alleles separately, we found a highly significant difference in LB number between donors carrying an *ε*4 allele and donors not carrying this allele (*P* = 0.002). Surprisingly, the LB counts also correlated negatively with GFAP levels in CSF (*r* = −0.385; *P* = 0.033). We did not find further correlations between LBs and clinicopathological data, such as sex (*P* = 0.322), PMD (*P* = 0.065), and amyloid score (*P* = 0.659).

## 4. Discussion

We have shown that astrogliosis in the frontal cortex of PDD and DLB patients is not increased compared to PD patients. In addition, we showed that there is no correlation between GFAP and vimentin levels in CSF and the cognitive status in these three LB disorders. Therefore, astrogliosis, as demonstrated by GFAP levels and the number of vimentin-positive astrocytes, does not seem to contribute to the frontal-type dementia in PDD and DLB. Furthermore, we showed that cortical LB pathology is not associated with an increase in cortical astrogliosis in PD, PDD, and LBD patients. However, we could confirm the earlier reported correlation of an increase in GFAP and vimentin in grey matter with increasing age and the association of *α*-synuclein pathology in the frontal cortex with LB-related dementia.

The increase in GFAP and vimentin in the aging brain is in agreement with previous studies showing a gradual increase of GFAP in astrocytes throughout the adult lifespan of mice, rats, and humans [35, 36]. While astrocyte cell size and processes increase with age, the total number of astrocytes seems to be unchanged [37]. The cause of the increase in reactive astrogliosis in the aged brain is unknown, but it has been proposed that it may be a response to oxidative stress and inflammation in the aging brain [38]. In contrast to previous studies in rodents [39, 40], no correlation between astrogliosis in cortical white matter and age was found. This discrepancy might be caused by species differences between human astrocytes and rodent astrocytes [41]. The difference between grey and white matter astrocytes may relate to the presence of neuronal cell bodies in grey matter. If astrogliosis is indeed a response to neuronal damage [42], it seems logical that grey matter astrocytes will respond more to aging. Vimentin is not exclusively expressed by astrocytes in the brain. In our study, we also observed a strong vimentin signal in the capillaries; therefore, we could not perform an automated image analysis method to measure the vimentin intensity. Next to this, also microglia express vimentin [43], and microglia have been observed to respond to *α*-synuclein accumulations in the substantia nigra of PD patients [44]. Since the vimentin expression pattern followed that of GFAP and since the morphology of the GFAP-positive cells was highly similar to the vimentin positive cells and reminiscent of astrocytes, we do not expect that potentially vimentin positive microglia affect our data. Our study showed that GFAP and vimentin levels in postmortem CSF correlated well, which suggests that levels of these proteins in CSF accurately reflect astrogliosis. We could detect vimentin in CSF only in a subset of donors, suggesting that vimentin protein is less stable than GFAP. There was no distinct group of donors that expressed vimentin in CSF, which indicates that the stability is not dependent on disease. Furthermore, there was no correlation with postmortem delay or pH of the CSF. Surprisingly, we did not find a correlation between astrocytic protein levels in CSF and astrogliosis in the frontal cortex. This might indicate that ventricular CSF levels of astrocytic proteins reflect astrogliosis in other areas, probably closer to the ventricle. There is evidence that protein levels vary in CSF samples from different areas, that is, ventricular versus lumbar CSF [45] and lumbar versus cisternal CSF [46]. It seems logical then that CSF from different compartments differently reflect tissue levels of these proteins.

The levels of GFAP and vimentin in CSF did not correlate with the severity of the cognitive impairment in PDD or DLB. There were also no differences in CSF-GFAP levels between the four diagnostic groups, showing that GFAP in CSF cannot be used as an indicator for astrogliosis or dementia in PD. These findings are in agreement with previous studies on PD CSF from lumbar punctures [47, 48]. We now describe that vimentin levels in CSF show the same lack of correlations, making it equally unsuitable as a biomarker for PD or dementia in PD. Levels of GFAP in CSF of AD cases are elevated and correlate to the severity of dementia [15], which is in contrast with our findings in PD cases. Therefore, we suggest that the pathological process underlying dementia in these two neurological disorders is different. This might be related to the nature of the aggregates in both diseases; LBs are found intracellularly, whereas A*β* accumulates extracellularly, thereby triggering astrogliosis. We did find that GFAP was expressed highly in CSF in a PD donor that died after a stroke, which is in accordance with an earlier report [49]. Vimentin expression was not remarkably high in this patient, which is surprising, since vimentin is upregulated in tissue from stroke patients [50].

Our finding that LB pathology correlates with cognitive parameters in LB-related dementias is in agreement with two previous studies. One of these studies identified LB load in the frontal cortex as the most significant indicator of cognitive impairment in PD patients, as measured by the GDS scale [27]. A later study reported on a correlation between frontal LBs and cognition and identified the entorhinal and anterior cingulate cortex as better predictors [51]. For DLB, no correlation was found between cortical LB pathology and dementia severity [52]. We find a similar number of frontal LBs in PDD and DLB, which is in accordance with the current thought that cortical LB pathology cannot be used to differentiate between DLB, PD, or PDD [53].

Aggregates of *α*-synuclein have not only been observed in Lewy neurites and in neuronal Lewy bodies but also in the cytoplasm of astrocytes [21, 34]. These findings contributed to the novel idea that glia dysfunction is involved in the pathogenesis of PD and other *α*-synucleinopathies. The accumulation of *α*-synuclein in astrocytes is proposed to be involved in initiating a long-lasting microglia response and reactive gliosis leading to neuronal degeneration [54, 55]. In our study, we did not discriminate between glial and neuronal *α*-synuclein inclusions. Unexpectedly, however, we observed that the number of LBs negatively correlated with GFAP levels in CSF. This finding underscores that *α*-synuclein does not lead to astrogliosis, and that *α*-synuclein might even reduce the reactivity of astrocytes [21], which might explain the decrease in GFAP levels in the CSF in patients with a higher number of LBs.

 The association we have found between the ApoE genotype and *α*-synuclein-positive LBs has been suggested before. Parkinson patients have been reported to have an overrepresentation of the *ε*2 [56] or of the *ε*4 [57] allele. An extensive meta-analysis identified a correlation between the ApoE *ε*4 allele and dementia in PD [56], but other studies have disputed this association [57, 58]. These mixed results may be a consequence of the variable amount of Alzheimer's pathology present in the different patient groups. We did not find an association between ApoE genotype and clinical phenotype, but we did observe more cortical *α*-synuclein pathology in carriers of the *ε*4 allele, which is in agreement with a previous study [27]. What these findings mean for the aetiology and disease progression of PD and PDD remains to be elucidated. One study has shown, using transgenic mice overexpressing mutant *α*-synuclein, that *α*-synuclein-induced neurodegeneration involves ApoE upregulation, and that ApoE further contributes to the disease process. In addition, amyloid beta was accumulating in this model, and the ubiquitin-proteasome system was disturbed, suggesting a common pathway between PD and AD [59]. The involvement of ApoE in PD and/or PDD is an indication that astrocytes are involved in the disease, since ApoE is expressed primarily by astrocytes [60]. Again, how this relates to the aetiology of PD and PDD needs further examination.

 We did not find a difference in astrogliosis or LB pathology in the frontal cortex between PDD and DLB. Currently, these two disorders are considered to be two clinical phenotypes of the same disease spectrum, and no markers have been found to distinguish the two conditions (reviewed in [61]). It is also not possible to make a distinction between the two phenotypes based on neuropsychological tests (reviewed in [62]). We found that the GDS score was on average higher in DLB cases than in PDD cases, but our results may be biased because we investigated the end stage of the disease. A factor that may distinguish between PDD and DLB is *β*-amyloid (A*β*). Striatal A*β* pathology is higher in DLB than in PDD [63, 64]. In addition, cortical A*β* load is significantly higher in DLB than in PDD, which could be measured in a PET scanner [65]. In our study, we did not find a correlation between amyloid scores and dementia or increased amyloid scores in DLB versus PDD, but we selected the cases for early Alzheimer stages, which might skew our data.

In summary, in the present study we could confirm that LB pathology in the frontal cortex was associated with dementia in LB-related dementias. However, we could not find any indication for an increase in astrogliosis in cortical areas in PDD and DLB brains or for a correlation between the extent of cortical astrogliosis and dementia. Furthermore, we showed that the presence of GFAP or vimentin in CSF could not be used as an indicator of astrogliosis or dementia. In conclusion, reactive gliosis is not associated with the cognitive impairment in LB-related dementias, in contrast to what has been reported for AD.

## Figures and Tables

**Table 1:** Clinicopathological data per group.

Diagnosis	Sex (M/F)	Age (yrs)	PMD (hrs: min)	Brain weight (gr.)	Age of onset (yrs)	Disease duration (yrs)	GDS	PD Braak stage
Control	7/3	82	06:07	1350	—	—	n.d.	0 (*n* = 6)
PD	7/2	79	05:35	1289	64	15	n.d.	5.0 (*n* = 5)
PDD	7/4	76	05:50	1276	69	10	5 (*n* = 7)	6.0 (*n* = 8)
DLB	7/3	75	04:20	1211	66	7	7 (*n* = 10)	5.0 (*n* = 2)

Median values are given; Control: nondemented control; PD: Parkinson's disease; PDD: PD with dementia; DLB: dementia with Lewy bodies; M: males; F: females; PMD: postmortem delay; GDS: global deterioration scale [16]; n.d.: not determined. There is no significant difference between the age (*P* = 0.301), the brain weight (*P* = 0.284), and the postmortem delay (PMD; *P* = 0.229) of the groups. The mean GDS scores of the DLB group significantly higher than of the PDD group (*P* = 0.004). All other donor parameters, such as amyloid pathology and ApoE genotype (see also Tables 1 and 2), were not significantly different between the groups.

**Table 2:** Clinicopathological data of individual donors.

NBB number	Diagnosis	IHC	CSF	Sex	Age (yrs)	Age of onset (yrs)	Disease duration (yrs)	PMD (hr: min)	pH CSF	Brain weight (gr.)	ApoE	GDS	Braak stage (Tau/amyloid)	Braak stage PD	Cause of death
1988-109	DLB	+	−	M	78	?	?	03:15	6.77	1240	33		1 B		Angina pectoris
1992-055	DLB	−	+	M	72	64	8	04:15	6.77	1423	32	7	1		Aspiration pneumonia
1995-109	PD	+	+	M	70	66	4	05:35	6.6	1602	32		1		Pneumonitis
1997-064	PD	−	+	M	65	39	26	06:00	6.35	1319	33		1		Sepsis after urinary tract infection
1997-092	DLB	+	+	F	79	77	2	03:20	6.4	1011	43	7	1	5	Influenza
1997-126	PD	−	+	F	83	78	5	04:40	6.16	1084	33		1	5	Natural death
1997-138	DLB	+	+	M	74	70	4	04:15	6.12	1093	43	7	1	5	Cachexia
1998-043	PD	+	+	F	81	64	17	04:10	6.86	1330	32		1		Pneumonia
1998-088	PDD	+	+	M	76	66	10	05:15	6.64	1283	32	4	1	5	Pneumonia
1999-038	PDD	+	+	F	86	71	15	06:00	6.34	1103	33		1 A	5	Dehydration
2000-015	C	+	+	M	78	—	—	05:35	6.63	1467	43		1 O		Thrombus leg/respiratory insufficiency
2000-115	PDD	+	+	M	70	53	17	09:05	6.33	1258	33	3	1 O		Pneumonia/septic shock
2001-025	DLB	+	+	F	76	66	10	04:05	6.52	1145	44	7	1 B		Dehydration
2001-026	DLB	+	+	F	72	65	7	07:25	7.64	1211	33	7	1 A		Dehydration
2001-037	DLB	+	+	M	80	78	2	06:40	6.47	1426	33	7	1 B		General deterioration
2001-045	C	+	+	M	83	—	—	04:35	6.49	1422	33		1 B	0	Heart attack
2001-046	C	+	+	M	88	—	—	07:25	6.5	1228	33		1 C	0	Metastasized prostate carcinoma (patient requested euthanasia*)
2001-104	C	+	−	F	77	—	—	05:30	6.74	1343	43		1 B		Lung metastases from Mammacarcinoma
2001-122	PD	+	+	M	86	60	26	05:35	6.19	1244	33		1 O	4	Aspiration pneumonia
2002-017	DLB	+	+	M	72	60	12	06:50	6.32	1270	43	6	1 A		Dehydration
2002-024	C	−	+	F	75	—	—	05:30	7.2	1197	42		1 C	0	Heart attack
2002-048	DLB	+	+	M	70	63	7	04:25	6.56	1211	43	6	1 A		Cachexia
2002-087	C	+	+	M	71	—	—	07:40	6.2	1190	33		1 O		Sepsis
2002-105	PD	+	+	M	83	77	6	06:20	6.71	1475	33		1 A		Cerebrovascular accident
2003-059	PDD	+	+	F	75	71	4	06:10	6.38	1258	43		1 A		Dementia/dehydration/fever
2003-078	PDD	+	+	F	61	53	8	04:30	6.3	1134	43		1 B		Abdominal problems
2004-033	PDD	+	+	M	73	69	4	05:35	6.8	1322	42		1 A	5	Unknown
2004-045	PDD	+	+	M	71	59	12	06:58	6.55	1358	43	6	1 C	6	Pneumonia
2004-057	C	+	−	F	81	—	—	06:40	7.16	1164	33		1 B	0	Metastasized cholangiocarcinoma (patient requested euthanasia**)
2004-059	PD	+	+	M	74	53	21	02:50	6.55	1259	33		1 O	5	Dehydration/uremic coma
2004-081	PDD	+	+	M	88	78	10	05:50	6.66	1205	32	2	1 C	6	Unknown
2005-017	C	+	+	M	87	—	—	10:20	6.32	1356	33		1 A	2	Pneumonia/heart failure
2005-050	PD	+	+	M	84	82	2	06:05	6.42	1243	43		1 C	5	Heart attack
2005-055	C	+	+	M	84	—	—	07:05	5.9	1385	33		1 O		Exacerbation of COPD
2005-069	PD	+	+	M	77	64	13	08:05	6.45	1380	32		1 O	5	Pneumonia leading to septic shock
2005-077	PDD	+	+	M	83	81	2	05:15	6.66	1276	33	6	1 B	6	Pneumonia
2006-030	PDD	+	+	F	80	68	12	05:15	6.31	1288	43	7	1 C		Dehydration with general deterioration
2007-007	C	+	+	M	84	—	—	05:35	6.98	1457	33		1 A	0	Heart failure
2007-008	PDD	+	+	M	80	74	6	07:05	6.34	1612	43	5	1 B	6	Unknown
2007-019	DLB	+	+	M	86	82	4	06:45	6.2	1180	43	7	1 B		Dehydration/cachexia after TIA

NBB: Netherlands Brain Bank; C: nondemented control; PD; Parkinson's disease; PDD: PD with dementia; DLB: dementia with Lewy bodies; IHC: immunohistochemistry investigated; CSF: cerebrospinal fluid investigated; M: male; F: female; ApoE: ApoE genotype; GDS: global deterioration scale [16]; Braak stage is a scale for Alzheimer's pathology, scoring tau [28], and amyloid [29] pathology; PMD: postmortem delay; pH CSF indicates the agonal state of the donor [30]; COPD: chronic obstructive pulmonary disease; TIA: transient ischemic attack; *euthanasia is legal according to Dutch law, the patient died of a combination of fentanyl, midazolam, thiopental, and pancuronium; **patient died of a combination of thiopental and pancuronium.

**Table 3:** Median results per group.

Diagnosis	GFAP GM (surface %)	GFAP WM (surface %)	Vimentin GM (cells per mm^3^)	Vimentin WM (cells per mm^3^)	GFAP CSF	Vimentin CSF	Lewy bodies (per mm^3^)
Control	1.22	1.37	963.3	3123	2.76	0	0.0
PD	0.99	0.92	761.2	1336	11.62	1.17	1.1
PDD	1.13	1.27	705.6	2533	1.98	0	29.8
DLB	1.12	1.00	1084.0	1965	4.36	0	19.2

Control: nondemented control; PD: Parkinson's disease; PDD: PD with dementia; DLB: dementia with Lewy bodies; GM: grey matter; WM: white matter; CSF: cerebrospinal fluid.
